# Integration of Innovative Technologies in the Agri-Food Sector: The Fundamentals and Practical Case of DNA-Based Traceability of Olives from Fruit to Oil

**DOI:** 10.3390/plants11091230

**Published:** 2022-05-02

**Authors:** Rayda Ben Ayed, Mohsen Hanana, Sezai Ercisli, Rohini Karunakaran, Ahmed Rebai, Fabienne Moreau

**Affiliations:** 1 Laboratory of Molecular and Cellular Screening Processes, Centre of Biotechnology of Sfax, P.B. 1177, Sfax 3018, Tunisia; raydabenayed@yahoo.fr (R.B.A.); ahmed.rebai@cbs.rnrt.tn (A.R.); 2 Laboratory of Extremophile Plants, Centre of Biotechnology of Borj-Cédria, B.P. 901, Hammam Lif 2050, Tunisia; mohsen.hnana@cbbc.rnrt.tn; 3 Department of Horticulture, Faculty of Agriculture, Ataturk University, 25240 Erzurum, Turkey; sercisli@gmail.com; 4 Unit of Biochemistry, Faculty of Medicine, AIMST University, 08100 Bedong, Malaysia; 5 Department of Computational Biology, Institute of Bioinformatics, Saveetha School of Engineering (SSE), Saveetha Institute of Medical and Technical Sciences (SIMATS), Thandalam, Chennai, Tamil Nadu 602105, India; 6 Centre of Excellence for Biomaterials Science, AIMST University, Bedong 08100, Malaysia; 7 Institut National de la Recherche Agronomique (INRA), 2 Place Pierre Viala, 34000 Montpellier, France; fabienne.moreau@qualtech-groupe.com

**Keywords:** artificial intelligence, big data, blockchain, DNA technologies, internet of things, smart agriculture, olive fruit

## Abstract

Several socio-economic problems have been hidden by the COVID-19 pandemic crisis. Particularly, the agricultural and food industrial sectors have been harshly affected by this devastating disease. Moreover, with the worldwide population increase and the agricultural production technologies being inefficient or obsolete, there is a great need to find new and successful ways to fulfill the increasing food demand. A new era of agriculture and food industry is forthcoming, with revolutionary concepts, processes and technologies, referred to as Agri-food 4.0, which enables the next level of agri-food production and trade. In addition, consumers are becoming more and more aware about the origin, traceability, healthy and high-quality of agri-food products. The integration of new process of production and data management is a mandatory step to meet consumer and market requirements. DNA traceability may provide strong approach to certify and authenticate healthy food products, particularly for olive oil. With this approach, the origin and authenticity of products are confirmed by the means of unique nucleic acid sequences. Selected tools, methods and technologies involved in and contributing to the advance of the agri-food sector are presented and discussed in this paper. Moreover, the application of DNA traceability as an innovative approach to authenticate olive products is reported in this paper as an application and promising case of smart agriculture.

## 1. Introduction

By 2050, the worldwide population is expected to reach over 9 billion, implying a significant increase in food needs and, therefore, a higher agri-food industry production [1]. The latest developments should be integrated and more accurate data should be better authenticated through new biotechnology in order to fulfill food production requirements [2]. Information sensing, saving, integration and automated analysis will help in making faster and more efficient decisions. In this regard, the Food and Agriculture Organization of the United Nations (FAO) focuses on how global agri-food production systems should change and evolve in order to support the subsistence needs of the worldwide population [3]. The essential components of agriculture and food supply chain are tightly connected to one another, because agricultural products are very often used as inputs in several multi-actor distribution supply chains, where the consumer is usually the final end user or client [4]. The new concept for agri-food supply chains designed as a “from field to fork” approach consists of connecting events in the agri-food production, where its process is made of a succession of chain of elements from production to processing, trading, distribution and consumption. Over the last decades, several and diverse crises and food safety incidents impaired the food sector [5,6], such as dioxin in poultry [7], bovine spongiform encephalopathy (well known as mad cow disease) [8], the threat of antibiotic-resistant pathogens [5], contaminated powdered milk [6] and the controversy over genetically modified organisms [9,10,11,12]. Hence, valuable and important reviews have been already written about Agri-food 4.0 [2,13,14,15]. Moreover, the recent COVID-19 pandemic affected and enhanced the already existing world economic crisis [16,17,18]. Lockdowns and intermittent work exacerbated the problems of farming activities and the entire food supply chain. Indeed, this unprecedented worldwide health crisis imposed restrictions and limitations on both the medical and food trade supply [18,19,20,21]. Although the availability of the COVID-19 vaccine in the world is currently quite limited, food and agricultural workers should be among the first to be vaccinated in order to protect themselves and their community, and to preserve the sustainability of the sector [22]. In addition, the agricultural sector is particularly suffering from the world health and economic crisis [22]. Hence, consumers’ confidence towards food safety is lacking. Therefore, the need for more information on agri-food products “from farm to fork” is increasingly demanded by consumers. In addition, among the recommendations made by OECD to build a resilient recovery and emerge stronger from the COVID-19 pandemic is the necessity to boost confidence in trade and global markets by improving transparency regarding trade-related policy actions and intentions [23]. In order to build trust, transparency and, consequently, to improve the agri-food value chain management in major features, such as manufacturing, traceability, information security and sustainable water management, and also in order to assemble agri-food production, it is crucial to adopt and apply, in each phase (production, processing, distribution, retailing and consumption) of the agri-food supply chain, the most advanced and latest technologies. This is why a well-documented traceability system has become a requirement for quality control in the food chain. The key challenges of the agri-food supply chain stakeholders, in the expected events for the coming years, are to increase revenues and, in return, to decrease the pressure of handling complex and external factors beyond their control, for example, rules and policies, market performance, weather and climate change conditions, but also to react on time by visualizing current trends in needs. In this worldwide socio-economic context and facing the COVID-19 pandemic scenario, the main objective of this review is to shed light on how, fundamentally, the exploitation of advanced technologies, referred to as Agri-food 4.0, could be helpful, and practically, the case of olive oil traceability by means of DNA markers could be successful.

## 2. Agri-Food 4.0

**Figure 1:**
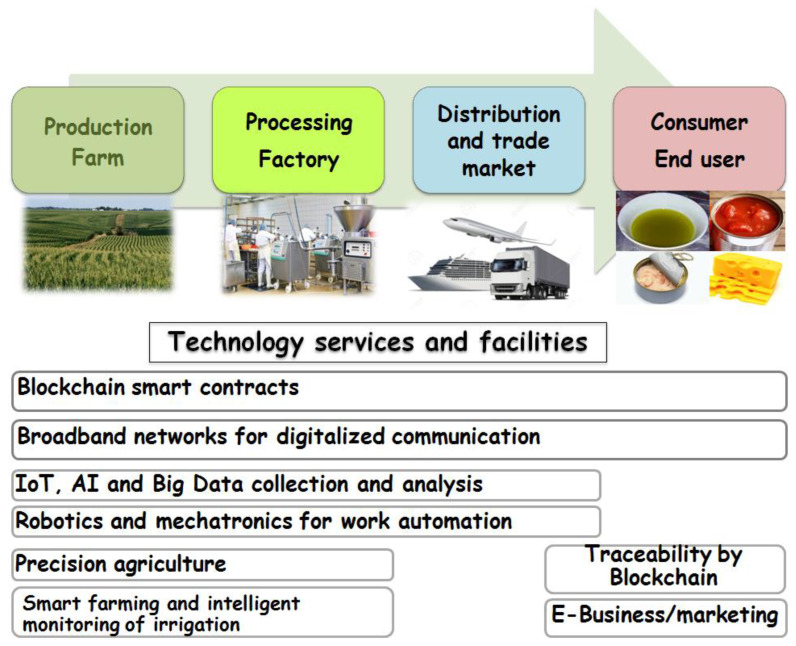
Smart technology integration in agri-food chains.

All the processes and events from agricultural production to food end product obtaining, including trading and supply chain, are called agri-food industry [2]. The development of agriculture from a state of primitive self-sufficiency to a capitalistic highly complex agricultural organization and the progress in the techniques have undergone several crucial steps [24]. Historically, the earliest beginnings of agriculture were mainly based on human force and animal traction. Starting from the 16th to the 19th century, mechanization, steam power and traction engines took place, corresponding to the first agri-industrial revolution [13,25]. The introduction of the Taylorism and Fordism concepts in the early 1900s, with the new labor organization, manufacturing management and standardization of work [26], together with the empowered engines, new machines, crops and farming techniques, led to the second industrial revolution at the beginning of the 20th century [25]. The advances in precision agriculture, robots, remote sensing and the management of farm information systems in the context of digital innovation started to appear two decades ago in the beginning of the 21st century, corresponding to the Agri-food 3.0 era [13]. Following the COVID-19 pandemic crisis, recently developed technologies, such as big data, blockchain and Artificial Intelligence, have been used and integrated to make agriculture smart and autonomous, connecting all the steps of food chain recess operations up to the end users. From hunting and gathering in early times to the industrialization, mechanization, automation, innovation and digitalization of agriculture, a new revolution in agriculture and food industry has begun the era of “Agri-food 4.0” (Figure 1).

### Agri-Food 4.0 Technologies

The management and the exploitation of big data in the agricultural sector are key nodes between agricultural concerns and digital transformation capabilities. In fact, the major issues concern the consolidation of open data, such as weather, soil and nutrients, governance data, such as local regulation, and data from end users or consumers with a referential data.

Among the recent advanced technologies are:

**Internet of things (IoT):** The term “Internet of Things” was invented by Kevin Ashton in 1999 during his work in supply chain optimization in order to promote radio frequency identification technology, as a new form of wireless communication [27]. According to McKinsey, the IoT is defined as sensors and actuators embedded in physical objects that are linked through wired and wireless networks, often using the same Internet Protocol that connects the Internet [28]. The IoT is a system of interrelated computing devices, mechanical and digital machines that are provided with unique identifiers (UIDs) and the ability to transfer data over a network without requiring human-to-human or human-to-computer interactions. In the agri-food sector, IoT technologies appear under labels such as “precision agriculture” or “smart farming” [29]. The technologies connected with the IoT have great potential for application in the sector of food and agriculture, but there are some societal, organizational and technological challenges faced by this field. Regarding societal challenges, the most important criterion is to convince all stakeholders in the food chain and to show them that using this technology provides significant benefits to society at several levels. Additionally, despite the lack of knowledge of technical skills by the end users, it is necessary to help them to learn and understand the utilization and applicability of these novel technologies by encouraging them to attend training sessions. As described in Table 1, there are numerous important challenges, such organizational and technological. In addition to tacking these challenges and to reduce to the bare bones the execution of the IoT in the entire supply chain, it is crucial to address several objectives of the agri-food sector in order to persuade users of the usefulness of these technologies in agriculture. The main objectives and benefits for farmers are the increase in yields, obtaining a better quality/quantity with lower production costs, the optimization of the process and respect of nature by reducing water consumption and other natural resources, and the improvement in the soil quality as well as the adaptation of crop management to the requirements of climate change by minimizing the ecological footprint and environmental impact of agricultural practices. Furthermore, these technologies enhance food safety and/or food security and ensure effective and fraud-free certification schemes (such as the organic labeling) across the entire food supply chain. In addition, it offers opportunities for new business and cooperation opportunities by developing business models personalized for an IoT environment.

IoT applications in agriculture are multiple and diverse, whether to make cultivation, livestock and fishing smart, or to increase the precision in farming through a global network system established by communications and interconnections between the network devices [30]. As for smart cultivation, drones could be used to optimize several farming processes using real-time data transmission from drone to the ground control platform [31]. With the assistance of IoT-based sensors, it is possible to monitor livestock better, and, for example, to localize and control the health and physical condition of livestock [31].

Regarding the olive oil industry, and through an Artificial Intelligence algorithm, the IoT was applied to determine the origin and traceability of Italian Extra Virgin Olive Oil (EVOO) in order to protect the ‘Made in Italy’ trademark [32]. As per the example, a multi-criteria evaluation method was developed in order to obtain a sustainable configuration of the Tunisian olive oil supply chain, synthesizing data and collecting information from agricultural production to oil extraction [33].

**Blockchain:** Blockchain is a digital transparent and secure transaction ledger technology that allows the storage and the transmission of the information. Although it is an emerging topic, it has been largely reported in the agricultural literature [34,35,36,37,38,39,40,41,42,43,44]. This new and innovative technological approach to realize decentralized trustless systems was developed in the year 2009 as the underlying platform for bitcoin exchange and has now evolved into a mainstream technology. The blockchain system uses a mixture of different tools that have been applied in computer science, for instance, in cryptography hash functions and database technology. There are three types of blockchain: open to all; private blockchain, whose access and use is limited to a certain number of actors; and the hybrid blockchain. The most well-known public blockchains, open to everyone, are the bitcoin blockchain and the Ethereum blockchain. Examples of private blockchains, whose access and use are limited to a number of players, are Hyperledger Fabric and Corda. Hybrid systems are also possible, where the protocol provides public access to the blockchain that serves as an infrastructure for the design of confidential private networks with authorization systems. Blockchain technology offers many advantages, including creating trust between two parties without an intermediate because the transmission of value between network users is based on asymmetric cryptography, drastically reducing transaction costs, particularly in the finance sector, and allowing the automation of immutable contracts. In practice, the blockchain ledger allows the implementation of “smart contracts” whose conditions are set in advance between two thirds, such as the public blockchain Ethereum, and another advantage of this technology is that it offers a new mode of governance by establishing a tamper-proof and ergonomic voting system. Moreover, this novel digital technology has the benefits of providing a coherent digital representation of physical assets and autonomous transaction executions as well as fault tolerance, immutability, secure, transparency and full traceability of the stored transaction records. This digital transaction ledger finds application in various fields. In fact, in order to make their business more efficient and transparent, many companies are venturing into the development of blockchain technology, especially in the fields of healthcare or finance. The application of this technology in the agri-food supply chain has been progressively expanded due to their benefits in this sector especially by providing an innovative solution to achieve food traceability, transparency, safety and security. Despite all its promises, blockchain technology has limits: firstly, a blockchain does not completely replace intermediaries; in fact, the smart contracts present on the blockchain are dependent on the entries manually introduced by the user and the user remains dependent on the platform that offers a user interface for this blockchain. Secondly, projects revolving around a blockchain are not infallible; indeed, despite the law code, there may be a malfunction, and the exploitation of a flaw by a hacker is a risk that should always be considered. Thirdly, some blockchains consume energy, such as the case of Proof of Work used by several blockchains, including Bitcoin; therefore, some initiatives have started to solve this problem by using the Proof of Capacity or the Proof of Stake. Finally, blockchain is theoretically not invulnerable to attacks. Blockchain technology has several revolutionary societal, business and governmental aspects, but, due to their weakness mentioned below, they might pose novel challenges that need to be predicted. Among these challenges, there are especially organizational, societal and technological problems, such as the large consumption of the energy, complex legal frameworks as well as uncertain regulatory status, lack of the needed technical skills and also the fact that blockchain requires the buy-in of its operators and users since this technology signifies a total shift to a decentralized network.

In the olive oil sector, several farmers have adopted the blockchain system and trusted its aptitude for tracing in the entire supply chain, from the plantation, farming techniques, production, packaging and conservation to the customer, while enabling olive oil traceability, extra virgin quality certification and most importantly providing a guarantee of quality and safety to the final consumers [45,46,47].

Finally, there are several technological challenges that should be anticipated:

Firstly, the infrastructure challenge by boosting and encouraging the availability of an Internet connection, which is still deficient in some developing countries. Secondly, the enhancement of the security and the control of the personal data, especially before it is addressed to the public. Thirdly, enlarging the capacity of the block size and the time interval used to generate a new block in order to rise the number of the transaction processes in real time. Fourthly, the modification of the transition strategy by changing or replacing the current system in order to offer the best solution by applying the blockchain system. Fifthly, and most importantly, a technological challenge is to make this digital tool a strength. It is important to rely on a suitable combination of different technologies (such as IoT and big data) with the purpose of improving the data-driven agri-food supply chain by making them more secure, efficient, informed, resilient and sustainable (Table 1).

**Big data (BD):** Big data refer to large, speed and complex data which are unable to be processed and managed by classical and traditional techniques [48]. These data are so vast, diverse and fast-changing that conventional technologies, tools and systems are not capable to address them efficiently [49]. Therefore, the big data term is also used to refer to technological tools and computer systems able to store, manage and process such big data [50]. In 2001, Gartner [51] defined “big data” as high-volume, -velocity and -variety information assets that demand cost-effective, innovative forms of information processing for enhanced insight and decision making [52]. More recently and according to the Big Data Value Association, volume, velocity, variety, veracity and value are the five ‘V’ keys making big data a vast business [13,53]. In fact, the five ‘Vs’ refer to the high volumes of low-density unstructured data, the fast velocity at which data are received and acted on, the variety of the availability of many types of data, level of trust and data quality and, finally, to detect exploited values from BD to support decision making. Several factors were at the origin of the appearance of the BD concept, especially the rapid generation of a large volume of data in 2005, after the emergence of numerous online services, such as Facebook. In addition, the advent of the Internet of Things (IoT) and machine learning produced more data. The greatest advantages of data analytics and BD are the possibility of users gaining more complete answers when they have more information, especially since BD provide more complete responses, so there is more confidence in the data. For these reasons, we characterize BD as a completely different approach used to tackle complex problems. Despite their benefits, BD have numerous general challenges. Firstly, every two years, the big data size is doubled, in spite of the development of new technologies for data storage. Moreover, industries and companies still struggle to keep up to speed with their data and discover the tools to efficiently stock them. Secondly, the challenge is not just stocking the data, but it is more important to curate the data well. In fact, data scientists spend 50 to 80% of their time preparing and curating data before their real use. Finally, because the big data approach changes at a rapid pace, technological tools must always be improved to handle big data. Over the past years, scientific research activity in BD technology began to provide pertinent results in several fields of application, such as marketing, manufacturing and healthcare. Recently, several technologies and tools have been identified and developed to continue research, for example, transformation algorithms, clustering methods, the management performance of data sources and storage.

Currently, the BD applied in the agri-food sector have received increased attention. In fact, previously, the storage, processing and analysis of information were impossible, owing to technological limitations. However, with the fourth industrial revolution and the apparition of the advanced technological and computational tools, BD management has become easier. In order to create new knowledge by extending farmer data and to generate innovative processes and services, the integration of BD technologies in agri-food projects has become crucial. Nowadays, there are numerous BD repositories that promise access to and the exploitation of agri-food data, i.e., OpenCorporates, NASA Earth Exchange, National Climatic Data Center, Soil Water and geospatial data from the National Resources Conservation Service (US). There are several positive impacts of the use of BD in the agri-food sector, such as functional (what should happen); predictive (what could happen); descriptive (what has happened); economic (enhance farm productivity by optimizing novel models by means of historical data, such as weather, soil, seed and fertilizer; improve decision making through business; the cheaper and faster delivery of high quality products and improve the operational efficiency in real time); business (develop a shorter supply chain, offer a new operating model, understand better the preference of the end users or consumers and solve problems more rapidly); social (transform the traditional skills-based agriculture into digital agriculture and decrease risks); environmental (better resource use and the minimization of waste); and technological (to deal with the five ‘V’). BD technology is still in the testing stage and its implementation in agri-food value chain management has a number of constraints that need to be tracked. Firstly, there are some technological challenges, especially those related to improving the capability of dealing with the five ‘V’ by developing new computational tools to solve the problem of storage capacity because data volumes are increasing exponentially, to better combine the data from different sources (i.e., IoT, social network and sensors), to enhance real-time data treatment, to guarantee reliability and excellence, to provide more data value, to support the data connectivity and to combine the three levels of analytics. The effectiveness and performance of the perspective simulated models depend on those of the descriptive and predictive analyses and, lastly, on accelerating solution innovation and development by opening technological service platforms. Secondly, there are many organizational challenges that we would take into account in the near future in order to improve the management of the agri-food supply chain, such as BD decentralization, enhancing BD control, especially when involving heterogeneous actors, increasing BD trust, security and privacy among actors and appropriately and correctly transferring data. Finally, concerning the social challenges, similar to all new technologies, their suitable employment requires the availability of human resources who have advanced knowledge and skills on BD analysis. Additionally, it is important to explore the ethical implication of BD technology in the agriculture and food sectors. As a final social challenge, it is important to encourage agri-food actors, either companies or individuals, to apply this BD tool in their agri-food supply chain by demonstrating to them the value of innovation compared to its costs.

**Artificial intelligence:** The history of AI started in the early nineteenth century, in 1920, when the term ‘robot’ was coined in a fiction play in which android hands substituted human workers [54]. However, the most significant example of this powerful technology is the defeat of Garry Casparov, the ten times world chess champion, by the deep blue chess computer program. Making computer, machine or robot intelligent and smart in a similar way to human thinking is the objective of artificial intelligence. AI refers to a panel of technologies based on electronic devices, computer systems and robots that perform functions, increasing and enhancing the acuity, speed, precision and efficiency of the user’s activity [55,56]. AI applied to technology should be able to identify things, recognize objects, analyze profiles, find solutions, make decisions, order actions, predict anomalies and learn and memorize for the subsequent steps in the supply chain. The main goals of AI are to generate smart and autonomous systems through the implementation of human-like intelligence in machines, and therefore, automated decisions and actions. Automation and precise farming practices have become an urgent need to meet the very fast increase in worldwide food needs [57]. Agriculture faces several constraints, abiotic and biotic, in addition to the COVID-19 crisis and socio-economic circumstances, which increase the vulnerability of food production and supply chains [22,55,56]. Indeed, since World War II, the global agricultural sector has faced great disturbances and disruptions, whether concerning food product supply, market stability or even changes in consumer behavior and preferences [58]. The integration of AI systems would greatly help to mitigate these risk factors, and therefore improve food security and achieve self-sufficiency, whilst reducing poverty, minimizing hunger and preserving natural resources [56]. Emerging technologies based on AI can contribute to enhance productivity and food supply chain efficiency, while optimizing farming and sustaining biodiversity.

A final technological challenge of AI technologies is the making of these clever tools strong, rather than the other digital approaches; it is important to trust in the correct combination and interconnection with the different other technologies (such as IoT) by enhancing IoT with machine learning techniques to analyze the data captured by the sensors in real time in the agricultural sector. Table 2 shows a myriad of AI applications in the agricultural sector.

In the case of olive oil, adulteration could be detected through chemical analyses, but it is an expensive cost and lengthy procedure. Therefore, in order to reduce both cost and time, machine-learning- and artificial-intelligence-based methods combined with fluorescence sensor, ultrasound approaches, dielectric/laser spectroscopy, or even electronic noise, were developed to control the quality and authentication of olive oil with high accuracy [59,60,61,62,63,64,65,66].

**Table 2:** Different kinds of recent AI applications in agriculture.

Sector	Application	Tool/Technology	References
**Farming**	Improvement of the resilience of agriculture production systems	Remote sensing, unmanned vehicle, unmanned aerial systems, next-generation sequencing, high throughput phenotyping	[67,68]
	Yield and productivity prediction	Field imaging, deep learning approach, biological and environmental indicators integration	[69,70,71]
	Intelligent control system for the determination of the watering time of turfgrass plants	Computer vision system, artificial neural network	[72]
	Grain crops: disease detection, grain quality, phenotyping	Computer vision, graphics processing unit, deep belief networks, artificial bee colony, extreme learning machine	[73,74]
	Energy consumption forecasting	Mathematical models	[75]
	Determining the precise work area of agriculture machinery	IoT	[76]
	In-field estimation of strawberry ripeness	Hyperspectral imaging, deep learning approach	[77]
	Weed management	Machine vision, graphics processing unit	[78,79]
	Crop evapotranspiration equations and modeling	Symbolic regression technique, artificial neural network, extreme learning machine, meteorological data	[80,81,82,83]
	Prediction of the hydration characteristics of wheat	Adaptive neuro-fuzzy inference system, artificial neural network	[84]
	Water detection on the Earth’s surface	Remote sensing technique, reflectometry data	[85]
	Optimization of irrigation monitoring, pesticides and herbicides applications	Automated robotic systems, machine learning algorithms, wireless sensor network	[86,87]
	Non-destructive determination of the soluble solids content of citrus	Near infrared transmittance technology, variable selection algorithm	[88]
	Disease diagnosis, detection and control (paddy crop, olive, grapevine, apple and orchards)	Automated vision, image processing, neural network architectures	[89,90,91,92,93,94]
	Irrigation water demand forecasting	Runoff simulation method	[95]
	Soil temperature assessment	Satellite imagery, regression-based models	[96]
	Health diagnosis of agriculture vehicles	Lightweight artificial intelligence technology, artificial neural networks, genetic algorithm	[97]
	Obstacle detection for agricultural machinery vehicle	Infrared binocular stereo vision system	[98]
	Perception of tractor implement performance in the plowing process	Soft computing workplace, non-linear equations	[99]
	Mechanical transplantation of pot seedlings	Robotics, mechatronics	[100]
	Crop damage avoidance during weed eradication	Mechatronics	[101]
	Citrus rootstock evaluation	Unmanned aerial vehicle-based remote sensing	[102]
	Detection of seed germination	Low-power embedded system	[103]
	Detection of post-harvest apple pesticide residues	Machine vision technology, AlexNet–CNN deep learning network	[104]
	Non-destructive detection of seed viability	Near infrared spectroscopy, infrared thermography, multispectral imaging	[105]
	Determination of the best drought tolerance indices	Artificial neural network	[106]
	Carbon sequestration and emissions mitigation in paddy fields	Denitrification–decomposition model	[107]
	Plant identification	Visual features of leaves, artificial neural network, support vector machine, algorithms	[108]
**Livestock monitoring**	Poultry monitoring	Computer vision systems	[109]
	Bird nest localization	Drone-borne thermal imaging	[110]
**Fishing/** **Aquaculture**	Separation of dead and live rainbow trout fish eggs	Visual machine technology-based intelligent system, imagery processing	[111]
	Fisheries management	Data collection and interpretation	[112]
**Forestry**	Spatial prediction of wildfire probability	neuro-fuzzy system and metaheuristic optimization algorithms, mathematical modeling	[113,114]
	Tree volume prediction	Machine learning	[115]
**Trade marketing**	Prediction of agricultural produce prices	Convolutional neural networks, artificial neural networks	[116,117]
	Input–output analysis of agricultural economic benefits	Big data	[118]
**Food industry and transformation**	Predicting optimum moisture content reduction in drying potato slices	Adaptive neuro fuzzy inference systems, artificial neural network, response surface methodology	[119]
	Modeling and optimization of *Terminalia catappa* L. kernel oil extraction	Artificial neural network	[120]
	Optimization of refrigerated transport	Computational fluid dynamics turbulence	[121]
	Applications for the food of electronic noses and tongues	Biosensors, chemical sensors	[122,123,124]
**Climatology**	Early warning of agricultural meteorological disasters	Big data	[125]

## 3. Advantages in Adopting DNA-Based Technologies in Agri-Food Supply Chain Traceability

The food industry is one of the most critical industries for human safety. However, in the future, food and agriculture will face numerous uncertainties, such as income distribution, climate change, population growth, technological progress, dietary choices, the sustainability of peace and the state of natural resources, especially since no one can know with precision the evolution of these factors over time and which certainly influence our future. Therefore, these factors pose serious problems concerning the performance and the sustainability of this vital sector. Consequently, it is time that researchers, international organizations and civil society increasingly perform an authoritative foresight exercise that outlines alternative scenarios and highlights potential pathways for food and agricultural systems. Because of these reasons, agri-food companies must invest more in a powerful and effective traceability system. In addition, in order to minimize risks and manage any issues quickly and efficiently, and also to achieve processing, managing and food product storing ensuring safety and quality assurance, it is crucial to propose an efficient traceability system. Multidisciplinary traceability, by combining advanced digital technologies (AI, blockchain, IoT) and DNA-based traceability, allows transparency and ensures the quality of services and products, to encourage the innovation of processes and products and to guarantee cognitive bases for decision making. To provide an unequivocal investigation of food authentication, to implement and to develop an effective and complete traceability system across the agri-food supply chain, advanced DNA-based analysis techniques are actually the most efficient approaches and are the subject of great interest (Figure 1). In the past, DNA examination techniques were used in different studies, such as the forensic and anthropology fields, whereas the application of this approach has been applied recently in the food production chain, especially after the appearance of several food safety scandals and incidents. The use of DNA-based methods in the field of food authenticity is gaining increasing attention and have been used to study the authenticity, traceability and adulteration of virgin olive oils [126], to test a variety of ground meat products sold on the U.S. commercial market for the presence of potential mislabeling [127], to identify and to authenticate fish products in Taiwan [128], to identify the potential toxicity of puffer fish species highlighting health concerns [129], and to verify cereal food composition and to detect adulterations [130]. However, in all these cases, both the quality and quantity of DNA extraction from complex matrices may represent a fundamental task in order to attain reliable and reproducible results.

### The Case of Olive Oil

Olive oil has become a highly desired product and its demand extends to all continents, especially extra virgin olive oil, whose quality fulfils strict international standards.

Nowadays, particular attention is paid to the study of the origin and authenticity of olive oil in order to gain the consumer’s confidence and to guarantee added value in terms of both quality and healthy products. The traceability of olive oils has therefore been developed and the subject of much research in recent years. It is an approach that involves tracing the different stages in the life of a product, from its creation to its consumption and destruction. Several techniques based on the analysis of the chemical composition of olive oil (fatty acids, polyphenols and antioxidants), such as gas or liquid chromatography and nuclear magnetic resonance (NMR), have been applied and introduced in the olive sector to study the authenticity and varietal origin of olive oils. Over the last decade, NMR has merged as a very attractive technique to record information about the origin and authenticity of food products [131,132], particularly olive oil traceability [133,134,135], showing high resolution and fidelity. However, it requires the use of isotopes such as ^13^C [136,137], ^31^P [138] and ^1^H [132,139,140], and is rather dedicated to the organic matrix of the non-volatile components of olive oil [133]. Moreover, NMR often needs to be coupled with other technique [140] and combined with chemometric methods and strong statistical analyses [141], which make it more a complementary approach than a real alternative for olive oil traceability and authentication [134].

In addition, several difficulties were encountered in distinguishing olive varieties, since their physico-chemical characteristics are strongly influenced by environmental conditions. Therefore, this analytical traceability of olive oil has proved to be insufficient due to its instability linked to the interaction with environmental factors. The technological progress associated with molecular biology has made possible to develop reliable and effective tools, particularly molecular markers based on DNA polymorphism. However, in order to succeed in the study of the traceability of olive oil, it is important to have efficient DNA that is extracted from the olive oil samples. With the possession of the knowledge that the task of DNA extraction from olive oil is very complicated, due to the characteristics of olive oil as a complex matrix, Ben Ayed et al. [126,142] not only succeeded in extracting an appropriate amount of DNA, but also obtained good quality residual DNA in order to provide regular PCR amplifications.

The molecular markers based on DNA polymorphism have shown a remarkable ability to study the traceability of olive oil. Different molecular markers have been applied (Table 3) as Random Amplified Polymorphic DNA (RAPD), Amplified Fragment Length Polymorphism (AFLP) and Simple Sequence Repeats (SSRs).

RAPD DNA markers are also used for the study of the authenticity and traceability of olive oil, but the researchers determined the non-reproducibility of RAPD markers in the authentication of olive oil, which resulted in an incompatible electrophoretic pattern.

The AFLP technique has been used to study the genetic structure of olives. This technique was also used to study genetic diversity. However, only 3 studies attempted to use the AFLP technique to study the traceability and authenticity of olive oil [151,154]. The results obtained were different, as Busconi et al. [154] reported that the AFLP profile of olive oil is partially superimposable with that of the variety from which the oil is extracted; on the other hand, the studies of Pafundo et al. [145] confirmed that the AFLP profiles of DNA purified from leaves and single variety oil of the same cultivar were comparable. This is probably due to the poor quality of the DNA, which is responsible for the inconsistent results and low reliability of the AFLP profiles due to the inhibition of restriction enzymes and DNA polymerase activity.

SSRs are excellent genetic markers applied in the study of the traceability of olive oil due to their numerous advantages, such as reproducibility, high polymorphism and discriminatory power, codominance and multi-allelic properties. In fact, SSR DNA-based markers have become available and reliable molecular markers to answer traceability questions, define the origin of olive oil and to detect the presence of undesirable varieties and fraudulent practices. Most of these publications also aimed to optimize the extraction of good quality DNA from olive oil and identify the most interesting SSR markers for variety discrimination.

**Figure 2:**
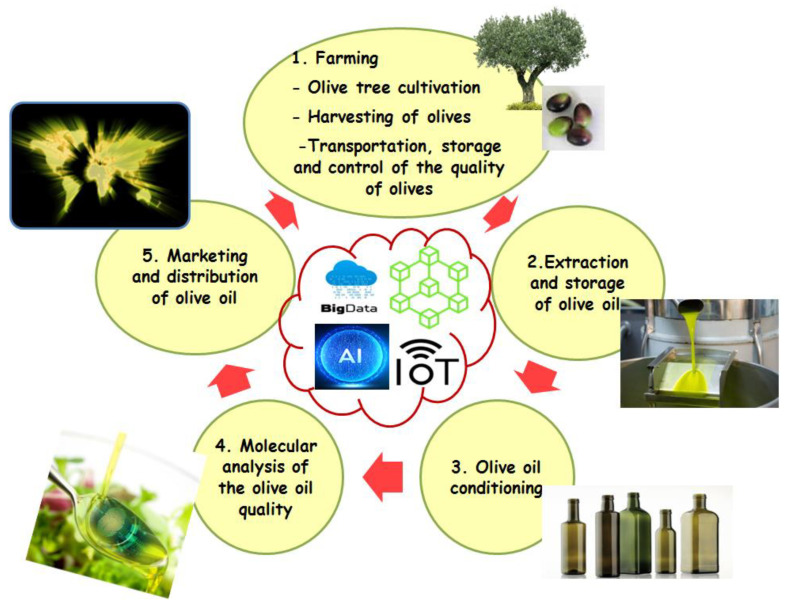
Integration of technology 4.0 in the olive oil supply chain.

In the case of the olive oil supply chain (Figure 2), Ben Ayed et al. [126] unveiled the DNA code present in trace amounts in the olive oil samples. They developed an innovative system based on multidisciplinary tools to study the traceability, authentication, adulteration and labeling of commercial olive oil. The results of this work allow for the accurate, rapid and cheap provision of the genetic and molecular identities of the product. This, in turn, will grant economic benefits to these olive oils through the application of this labeling strategy for their authentication. However, following the developments of new technologies (e.g., Blockchain, IoT, AI and BD) and biotechnological progress, these analyses, based on the analytical techniques, soon became outdated, hence the interest and the need to improve, develop and modernize them. To help alleviate these difficulties, the method for studying the traceability of olive oils can only be effective through a multidisciplinary approach involving several fields: molecular biology, genetics, plant biotechnology, bioinformatics and biostatistics. Moreover, the use of DNA molecule-based analyses in olive oil is a novel approach. The efficiency of this innovative tracking system requires the registration of the varieties and their characteristics in an organized and hierarchical way, and it is therefore necessary to have a database. Indeed, with the emergence of advanced computer tools, the conservation of the genotypes and characteristics of different olive varieties is becoming a necessity, and, in this context, Ben Ayed et al. [151] implemented the first database of olive genotypes, from all over the world, named the OGDD (Olive Genetic Diversity Database). This molecular multidisciplinary approach has successfully overcome the problem of the traceability of commercial monovarietal and multivarietal oils, and complemented the contribution of biochemical and organoleptic characteristics for the characterization and traceability of olive oils. In fact, the use of DNA-based technologies is of a great interest to meet the needs of consumers and will become essential for studying the traceability of olive oil, especially after the emergence of the COVID-19 pandemic. The implementation of the molecular DNA Label will not only allow for better authentication and a more reliable characterization of the added value, but, above all, it will engender a more trustworthy relationship in relation to the olive oil sector.

The utmost objective is to facilitate exports through the differentiation of the products by means of original and innovative scientific techniques. The particularity of this innovative system aims to establish strong authentication for the consumers and producers. Nowadays, the urgency related to the apparition of the COVID-19 pandemic requires targeted and intelligent interventions. Hence, these analyses occupy a strategic position in relation to trade, increase corporate income, improve access to the global competitive environment and win new markets. Due to their high variability, according to environmental conditions, the higher error percentage, neither the morphological characteristics of different the groups nor the analyses of the chemical composition of major and minor compounds can provide reliable results for olive oil traceability.

## 4. Conclusions, Vision and Key Advice

The impact of COVID-19 on the agriculture and food industry, together with the increase in the worldwide population and the growth of the agri-food market stimulate the innovation of technology in the field of production to fulfill the needs of the end user. The succeeding technologies involving IoT, blockchain, big data, artificial intelligence and molecular and nanotechnologies have the common aims of increasing system productivity, ensuring healthy products and optimizing trade markets and benefits, while ensuring sustainability and plant–soil preservation, and avoiding environmental pollution and degradation. Almost all the sectors of the agriculture and food industry have been concerned by this revolutionary approach, implementing innovate technologies and highly sophisticated equipment. The revolutionary devices and advanced technologies used in agriculture would increase productivity and income, save on cost production and energy, reduce labor and the expenditure of time and expand trade and the market; however, there are some disadvantages and drawbacks to this Agri-food 4.0 trend. Indeed, since this requires a constant Internet connection with a high flow, this could be problematic in rural regions and developing countries, in which the Internet connection is frustratingly slow. In addition, the smart farming approach requires a high level of technical skills and abilities that some farmers lack, or for whom they are expensive to acquire. Moreover, the excessive use of chemicals, whether for plant and animal nutrition, soil fertilization, or weeds and pest treatments, is opposed to sustainable agriculture. The mechanization and digitalization of the agri-food sector represent a serious risk of human job displacement. Agri-food production and industry still face challenges worldwide, climate change, a fast-growing global population and, most importantly, the COVID-19 crisis. Developing the agri-food sector with highly efficient and sustainable practices, combined with the next generation of molecular tools, is inevitable to meet the current challenges and future needs.

## Figures and Tables

**Table 1:** New technology challenges and constraints of the agri-food sector.

Technology	Challenges		
	Social	Organizational	Technological
**IoT**	- Persuade all stakeholders in the food chain using this technology that has a lot of benefits to either small or large farmers. - Help farmers to learn and understand the utilization and the applicability of these novel technologies.	- **Heterogeneity of the sector:** no single solution (business model, technological or regulatory) for the different types of actors in the food system can accommodate their needs. - **Variability of the farm sizes and capital investment costs:** the challenge is making the IoT offerings suitably appealing to small size farmers with restricted investment available for this technology. - **Business models and business confidentiality:** to retain control and ownership of farmer data by creating an Agricultural Data Coalition.	- **Lack of interoperability:** too many standards emerge. - **Lack of connectivity in rural regions:** Despite the existence of low power wide area (LPWA) technologies (for example, LoRa and SIGFOX) that provide a real opportunity to overcome such limitations, these technologies have the limitation that they cannot handle large datasets (originating from satellite imagery). The challenge is to improve connectivity with third, fourth or fifth generation coverage in order to more develop the IoT in agriculture. - **Data processing power:** the challenge is to set up a data processing service to help small-to-medium farmers to access a large-scale processing power at a reasonable cost for solving difficult calculations. - **Lack of clear data governance:** the challenge is to increase the control and the ownership of farm data. - **Data anonymity, security and privacy:** to adopt IoT in smart agriculture.
**Blockchain**	- Lack of the needed technical skills. - Blockchain requires the buy-in of its operators and users since this technology implies a total shift to a decentralized network.	- Large consumption of the energy. - Complex legal frameworks as well as uncertain regulatory status. - **Environmental challenges**	- The infrastructure is defiant by boosting and encouraging the availability of the Internet connection that is still deficient in some developing countries. - Enhancing the security and control of the personal data, especially before its address to the general public. - Enlarging the capacity of the block size and the time interval used to generate new blocks in order to rise the number of the transaction process in real time. - Modify the transition strategy by changing or replacing the current system in order to offer the best solution by applying the blockchain system. - To rely on a suitably combination of the different other technologies (such as IoT and big data).
**BD**	- The availability of human resources that advance knowledge and skills on BD analysis. - Explore the ethical implications of BD technology in the agriculture and food sectors. - Encouraging agri-food actors (either companies or individuals) to apply this BD tool in their agri-food supply chain.	- BD decentralization. - Enhancement of BD control. - Increase in BD trust, security and privacy among actors. - Transfer of data appropriately and correctly.	- Developing new computational tools to solve the problem of storage capacity because data volumes are increasing exponentially. - To better combine data from different sources (IoT, social network, sensors, etc.). - Enhance real-time data treatment. - Guarantee reliability and excellence. - Provide more data value. - Support data connectivity. - Combine the three levels of analytics (perspective, predictive and descriptive). - Open new technological platforms as a service to companies.
**AI**	- Increase in unemployment		- Lacking thinking out of the box: lack of flexibility and ability of machines to solve such problems, which are not programed or available in the algorithms designed. - High costs of creation and maintenance of the smart machines and cleaver computers. - Perform the correct combination and interconnection with the different other technologies (such as IoT) by enhancing IoT with machine learning techniques to analyze data captured by sensors in real time in agriculture.

**Table 3:** The DNA-based technologies for olive oil traceability.

DNA Marker Technologies	Advantages	Limits	References
**Random Amplified Polymorphic DNA** **(RAPD)**	The Polymerase Chain Reaction (PCR) is realized by using short primers (8–12 nucleotides) and amplifying random segments of DNA.	Very sensitive to DNA concentration and amplification conditions. Lack of reproducibility.	[143,144]
**Amplified Fragment Length Polymorphism (AFLP)**	AFLP is possible with a smaller quantity of the genomic template. As restriction sites are present across the entire genome of an individual, which allows the AFLP marker to analyze multiple loci at once.	Inconsistent results and unreliable AFLP profiles. AFLP cannot be performed with poor quality DNA or degraded DNA. As AFLP are dominant markers in nature, they cannot detect homozygous or heterozygous individuals.	[145]
**Simple Sequence Repeats (SSRs)**	Codominant, multi-allelic and highly abundant in the genomes of eukaryotes. High discriminatory power between the varieties. The results are highly reproducible, but this depends on the quality of DNA as the input.	The extraction of DNA from a liquid lipid matrix. The selection of appropriate molecular markers that can lead to significant results.	[126,142,143,144,145,146,147,148,149,150,151,152,153]

## Data Availability

All new research data were presented in this contribution.
